# Metabolomic characterization of human glioblastomas and patient plasma: a pilot study

**DOI:** 10.12688/f1000research.143642.1

**Published:** 2024-02-16

**Authors:** Yin Allison Liu, Orwa Aboud, Lina A. Dahabiyeh, Orin Bloch, Oliver Fiehn

**Affiliations:** 1 Department of Opthalmology, University of California Davis, Davis, California, USA; 2 Department of Neurology, University of California Davis, Davis, California, USA; 3 Department of Neurosurgery, University of California Davis, Davis, California, USA; 4 Comprehensive Cancer Center, University of California Davis, Davis, California, USA; 5 West Coast Metabolomics Center, University of California Davis, Davis, California, USA; 6 Department of Pharmaceutical Sciences, School of Pharmacy, The University of Jordan, Amman, Amman Governorate, Jordan

**Keywords:** glioblastoma, untargeted metabolomics, biomarker, feasibility, pilot

## Abstract

**Background:**

Glioblastoma (GBM) is a clinically challenging primary brain tumor with poor survival outcome despite surgical resection and intensive chemoradiation. The metabolic heterogeneity of GBM can become biomarkers for treatment response, resistance, and outcome prediction. The aim of the study is to investigate metabolic distinctions between primary and recurrent GBM tissue and patient plasma to establish feasibility for metabolic profiling.

**Methods:**

A single-center cohort study analyzed tissue and blood samples from 15 patients with GBM using untargeted metabolomic/lipidomic assays. Metabolomic, lipidomic, and biogenic amine analyses were conducted on GBM tissue and patient plasma at diagnosis and recurrence using untargeted mass spectrometry. The study utilized a small but longitudinally collected cohort to evaluate alteration in metabolites, lipids, and biogenic amines between specimens at diagnosis and recurrence.

**Results:**

Exploratory analysis revealed significant alteration in metabolites, lipids, and biogenic amines between diagnostic and recurrent states in both tumor and plasma specimens. Notable metabolites differed at recurrence, including N-alpha-methylhistamine, glycerol-3-phosphate, phosphocholine, and succinic acid in tissue, and indole-3-acetate, and urea in plasma. Principal component analysis revealed distinct metabolomic profiles between tumor tissue and patient plasma. Distinct metabolic profiles were observed in GBM tissue and patient plasma at recurrence, demonstrating the feasibility of using metabolomic methodologies for longitudinal studies. One patient exhibited a unique tumor resistance signature at diagnosis, possibly indicating a high-risk metabolomic phenotype.

**Conclusions:**

In this small cohort, the findings suggest the potential of metabolomic signatures of GBM tissue and patient plasma for risk stratification, outcome prediction, and the development of novel adjuvant metabolic-targeting therapies. The findings suggest metabolic discrepancies at diagnosis and recurrence in tissue and plasma, highlighting potential implications for evaluation of clinical response. The identification of significant changes in metabolite abundance emphasizes the need for larger studies using targeted metabolomics to validate and further explore these profiles.

## Introduction

Glioblastoma (GBM) is a fatal tumor with a median survival of less than two years. ^1^ Most GBMs respond to initial therapeutic interventions of surgical resection and chemoradiation ^2^^,^^3^ but eventually, all patients will relapse. The range of progression free survival is wide, from a few months to more than two years. Understanding the differences in molecular features of GBM and its microenvironment at diagnosis and at recurrence can help identify biomarkers to monitor for treatment response, understand pathogenesis of treatment resistance, and predict outcome. It may also help identifying new therapeutic targets.

Surgical resection and standard chemoradiation improve survival but this initial effect is limited by the development of resistance. ^4^ While therapy resistance and the aggressiveness of GBM have been investigated at the genomic and transcriptomic levels, less is known about the metabolic phenotypes. Altered metabolism is a hallmark of cancer. ^5^ Metabolomic changes are critical for tumor cells to undergo conversion to aggressive and treatment-resistant phenotypes. ^6^ Recurrent, therapy resistant tumors develop within the high dose radiation field, and the ability of recurrent tumors to resist therapy is, in part, due to metabolomic alternation within the tumor. ^7^ Tumor metabolism is influenced by both cancer cell-intrinsic information (genome, epigenome, proteome, post-translational modifications) and cell-extrinsic cues from the tumor microenvironment.

Targeting the metabolome has succeeded in a number of cancers including high-grade gliomas. ^7^ Glucose uptake can inform prognosis in a variety of cancers including glioma. ^8^ Metabolomic profiling in GBM tissue may provide important information on the differences in tumor responses to initial standard of care therapies and for understanding the differences between tumors at diagnosis and at recurrence. This may help predict tumor aggressiveness and patient prognosis. Early data from several metabolomic studies in small cohorts of patients with GBM have yielded promising results. ^9^^,^^10^ GBM is a heterotrophic tumor but is also known to have highly heterogeneous lipid metabolism ^11^ and favors heterotrophy. ^12^^,^^13^ However, the comprehensive metabolomic profile including metabolites, biogenic amines, and lipidomes have not been studied. The comparison between GBM tissue and patient blood specimens has not been performed. Therefore, it is unclear if patient blood can be used as a surrogate to predict tumor status in the brain.

We performed a study to test the hypothesis that recurrent GBMs are metabolically distinct from GBM at initial diagnosis, and patient plasma can be used as a liquid biopsy to reflect this difference. Using untargeted mass spectrometry, we profiled the metabolomes, lipidomes, and biogenic amines of human glioblastoma tissue and patient plasma both at diagnosis and at recurrence, and correlated metabolomic information with clinical data. We identified patterns of metabolomic remodeling in tumor tissue and patient plasma. These changes can pave the way for metabolomic signature identification for treatment response monitoring, risk stratification, and outcome prediction.

## Methods

### Ethical considerations

All subjects gave their written informed consent for inclusion before they participated in the study. The study was conducted in accordance with the Declaration of Helsinki, and the protocol was approved by the Ethics Committee of the University of California, Davis (Tissue collection project identification code 218204, original approval date 06/01/2005; plasma collection project identification code 1412052-2, approval date 4/16/2019).

### Glioblastoma tissue specimens

The UC Davis Pathology Biorepository at the Comprehensive Cancer Center provides high quality and well-characterized human brain tumor tissue specimens. In this centralized biorepository, all samples were collected after patients’ informed consents and underwent quality control by a clinical neuropathologist. From January 2010 to July 2022, a total of 12 fresh frozen GBM specimens were identified and obtained from the biorepository for metabolomic analysis. Limited deidentified clinical information was abstracted from the medical records within the scope of the approved IRB protocol of the biorepository. All patients underwent the standard Stupp protocol ^2^ for glioblastoma treatment.

### Plasma collection

The UC Davis Department of Neurosurgery (Dr. Orin Bloch Laboratory) has an IRB approved protocol to collect blood samples from patients with GBM at diagnosis and throughout treatment, along with access to clinical information of these patients. All procedures performed in this study were in accordance with the 1964 Helsinki Declaration and its later amendments or comparable ethical standards. From August 2019 to July 2022, a total of 12 plasma specimens were selected for metabolomic analysis.

All patients underwent the standard Stupp protocol ^2^ for glioblastoma treatment. Blood specimens were collected before surgery during the hospitalization. No anesthesia was administered at the time of the blood collection, but all patients were on high dose oral steroids in the peri-operative periods. Untargeted metabolomic, biogenic amine, and lipidomic analyses for GBM tissue and patient plasma were performed.

### Untargeted metabolomic, biogenic amine, and lipidomic analyses for GBM tissue and patient plasma

*Sample preparation and extraction*

Metabolites and biogenic amines were extracted as previously described. ^14^ Blood plasma or serum was extracted following the protocols first published by V. Matyash *et al.* ^15^ In detail, methanol (1.5 ml) was added to a 200 ml sample aliquot, which was placed into a glass tube with a Teflon-lined cap, and the tube was vortexed. Then, 5 ml of MTBE was added, and the mixture was incubated for 1 h at room temperature in a shaker. Phase separation was induced by adding 1.25 ml of MS-grade water. Upon 10 min of incubation at room temperature, the sample was centrifuged at 1,000 g for 10 min. The upper (organic) phase was collected, and the lower phase was reextracted with 2 ml of the solvent mixture, whose composition was equivalent to the expected composition of the upper phase [obtained by mixing MTBE/methanol/water (10:3:2.5, v/v/v) and collecting the upper phase]. Combined organic phases were dried in a vacuum centrifuge. To speed up sample drying, 200 ml of MS-grade methanol was added to the organic phase after 25 min of centrifugation. Extracted lipids were dissolved in 200 ml of CHCl3/methanol/water (60:30:4.5, v/v/v) for storage.

Using this protocol, lipid extracts in methyl tert-butyl ether phase (MTBA) were separated from proteins and polar hydrophilic small molecules (in the methanol/water phase) in a way that the lipids were found in the top layer of liquid-liquid separations, rather than in the bottom layer.

Decanting the top layer therefore ensured that the extracts were not contaminated by proteins or polar compounds. The top layer was used for lipidomics while the bottom layer (methanol/water phase) was very suitable for the hydrophilic interaction liquid chromatography-mass spectrometry (HILIC-MS) investigations.

*Data acquisition*

Metabolite profiling using HILIC-MS was performed on the Agilent 1290 UHPLC/Sciex TripleTOF 6600 mass spectrometer. Metabolites (5 μL) were separated using Waters AcquityUPLC BEH amide column (1.7 μm, 2.1 × 50 mm) and a binary mobile phase. Mobile phase A: 100% H _2_O +10 mM Ammonium Formate + 0.125% Formic acid; B: 95:5 ACN/H _2_O + 10 mM Ammonium Formate + 0.125% Formic acid. Chromatographic data acquisition was 15 min long. Data were acquired in data-dependent acquisition mode with a mass range 50-1,500 m/z for MS1 and 40-1,000 m/z for MS2.

Lipidomic data were acquired using the Agilent 1290 UHPLC/Agilent 6530 QTOF (for positive mode) and 6550 QTOF (for negative mode) mass spectrometer. Waters Acquity Premier BEH C18 column (1.7 μm, 2.1 × 50 mm) was used for chromatographic separation applying binary mobile phase system (Positive mode: mobile phase A: 60:40 v/v acetonitrile:water + 10 mM ammonium formate + 0.1% formic acid; mobile phase B: 90:10 v/v isopropanol:acetonitrile + 10 mM ammonium formate + 0.1% formic acid. Negative mode: mobile phase A: 60:40 v/v acetonitrile:water + 10 mM ammonium acetate; mobile phase B: 90:10 v/v isopropanol:acetonitrile + 10 mM ammonium acetate). MS scan range and mass resolution for positive mode were 120-1,200 *m/z* and 10,000, respectively, and 60-1,200 *m/z* and 20,000 for negative mode.

Primary metabolism data were acquired by gas chromatography (GC)-MS using an Agilent 7890A GC coupled to a Leco Pegasus HT TOF mass spectrometer. Extracts were dried down, derivatized by methoxyamination and trimethylsilylation, and injected in spitless mode with a temperature gradient from 50-330°C. Mass spectra were acquired at 17 Hz from 85-500 Da. ^14^

*Raw data processing and metabolite annotation*

Acquired raw LC-MS and LC-MS//MS data were processed using the following steps: Raw CSH-C18-TOF (for lipidomics) and HILIC-TTOF (for polar metabolites profiling) MS data were processed using MS-Dial (version 4.9). Data-independent MS/MS deconvolution was performed for comprehensive metabolome analysis. ^14^ Raw GC-TOF MS data files were processed using ChromaTOF and metabolomics BinBase database. ^14^ Peak heights were used for analysis given the strong correlation between extracted ion peak area and peak height across a wide range of concentrations. For very abundant peaks, ion saturation may occur, and in such cases, peak area may be a slightly better measure than peak height. However, in metabolomics, most compounds are low in abundance. Therefore, peak height is a more robust way to measure metabolite levels because the nose baseline has less impact ( *e.g.*, for peak start/end) on peak height compared to peak area.

### Statistical analysis

Descriptive statistics were used to characterize baseline patient and treatment characteristics. Individual metabolite abundance comparisons at diagnosis and at relapse were performed using GraphPad Prism 9 (version 9.5, San Diego, CA). MetaboAnalyst 5.0 (version5.0, McGill University, Montreal, QC, Canada) ^16^ was used to generate principal component analysis (PCA), score plots, heat maps and volcano plots. The processed peak heights with their annotation were imported to MetaboAnalyst, normalized to the total sample median and auto scaled. Paired or unpaired Student’s *t*-Test was used to identify significantly altered metabolites between the compared groups ( *p*-value of less than 0.05 was considered significant). ChemRICH (version 2023), a statistical enrichment approach based on chemical similarity rather than sparse biochemical knowledge annotations was used to group the metabolites. ChemRICH sets have a self-contained size where p-values do not rely on the size of a background database.

The protocols are deposited at Protocol IO: https://dx.doi.org/10.17504/protocols.io.n92ldmjkol5b/v1.

## Results

### Cohort description

Patient demographics are described in Table 1. ^29^ In the tissue cohort, a total of 12 specimens from nine patients were analyzed (nine specimens at diagnosis, three of which had paired specimens at recurrence). The mean age at diagnosis was 49 years old. There was a male predominance of 67%. Most of this cohort was non-Hispanic White in race/ethnicity.

**Table 1.** Patient characteristics. GBM, glioblastoma; IDH, Isocitrate Dehydrogenase; EGFR, epidermal growth factor receptor; MGMT, O ^6^-methylguanine-DNA methyltransferase; ATRX, Alpha thalassemia/mental retardation syndrome X-linked; PFS, progression-free survival; OS, overall survival.

Clinical and molecular features	Demographic profiles	
Total cohort n = 15	Tissue and plasma are not from paired patients	
GBM tissue, n = 9		
Age at diagnosis (years, range)	49 (31-60)	
Sex	Male	6 (67%)
	Female	3 (33%)
Race/Ethnicity	Non-Hispanic White	6 (67%)
	Hispanic/Latino	2 (22%)
	Not reported	1 (11%)
Plasma, n=6		
Age at diagnosis (years)	54 (43-60)	
Sex	Male	4 (67%)
	Female	2 (33%)
Race/Ethnicity	Non-Hispanic White	4 (67%)
	Hispanic/Latino	1 (17%)
	Black	1 (17%)
IDH	Wild type	6 (100%)
	Mutant	0
EGFR	Amplification	3 (50%)
	Negative	3 (50%)
MGMT	Methylated	3 (50%)
	Unmethylated	3 (50%)
ATRX	Retained	6 (100%)
PFS (months, range)	14 (6-24)	
OS (months, range)	17 (9-25)	

In the plasma cohort, a total of 12 paired specimens (diagnosis and at recurrence) from six patients with GBM were analyzed. The mean age was 54 years old. There was a male predominance of 67%. The original GBM tissue pathology all showed Isocitrate Dehydrogenase ( *IDH*) wild type. Additional pathology features including epidermal growth factor receptor ( *EGFR*), O ^6^-methylguanine-DNA methyl-transferase ( *MGMT*), and Alpha thalassemia/mental retardation syndrome X-linked ( *ATRX*) status are shown in Table 1. The mean progression free survival of this cohort was 14 months. The mean overall survival was 17 months.

### Unsupervised exploratory analysis on GBM tissue metabolomic, biogenic amine, and lipidomic profiling

**Figure 1.**
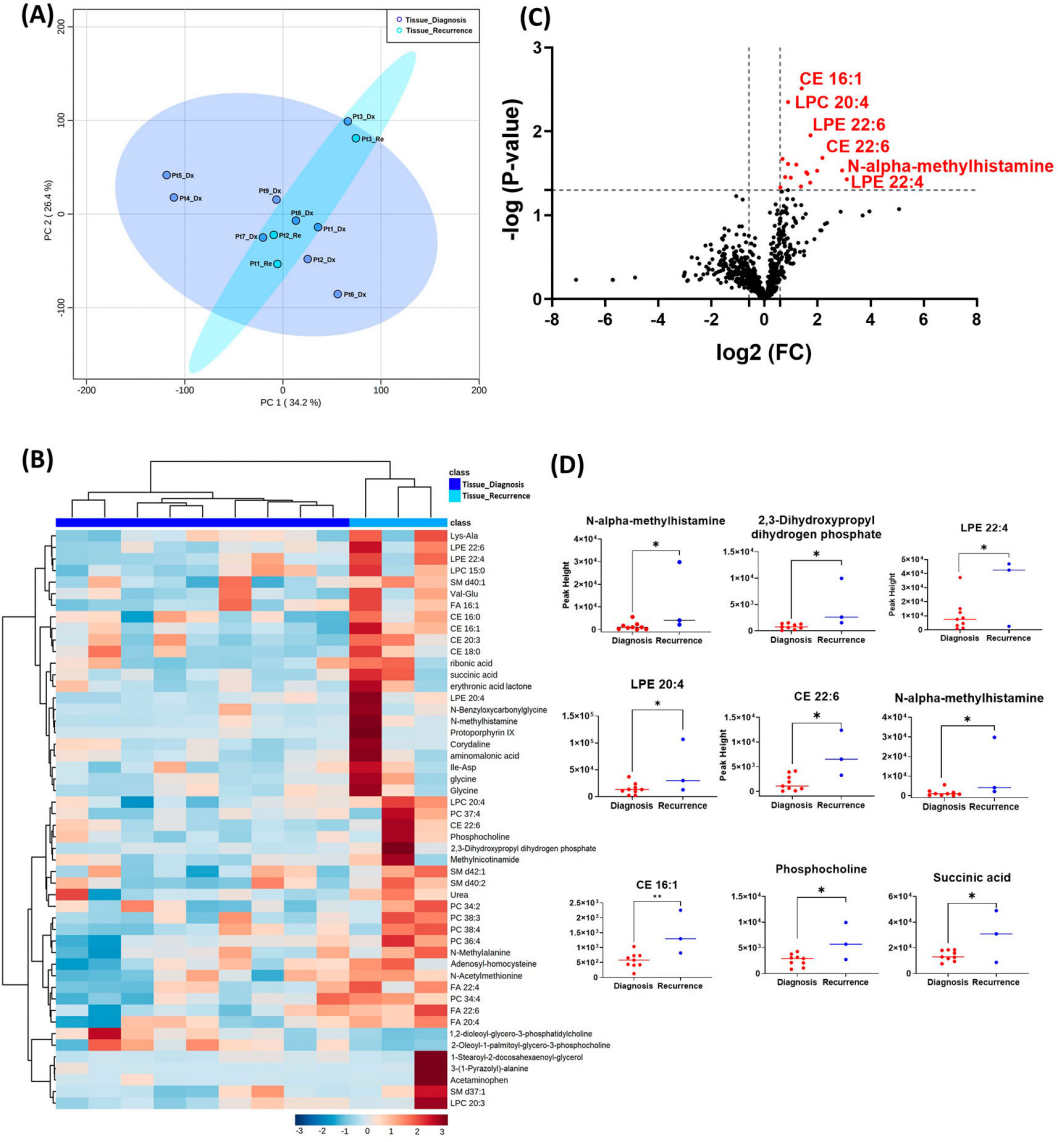
Comparison of metabolomic profiles of nine glioblastoma tumor tissue specimens at diagnosis and three tissue specimens at recurrence. (A) Principal component analysis (PCA) plot showing different trends of metabolomic characteristics between tissues at diagnosis (purple) and tissue at recurrence (light blue). (B) Heatmap of the top 50 altered metabolites at diagnosis and at recurrence. Blue indicates decreased peak value and maroon indicates increased peak value of each compound listed. (C) Volcano plot of up regulated metabolites in red and down regulated metabolites in blue in glioblastoma tumor tissue specimens at recurrence comparing to at diagnosis using p-value of <0.05 and fold change cutoffs of 1.5. (D) Plots of individual values for each metabolite demonstrating peak value changes at diagnosis and at recurrence in brain tumor tissue. Values were determined as peak heights from LC/MS analysis. Single asterisk indicates a p value of <0.05. Double asterisks indicate a p value of <0.01.

The GBM tissue cohort included nine tumor tissue specimens at diagnosis, three of which had paired tissue specimens at recurrence. A complete list of significantly altered metabolites is available in Table 2. A summary of these changes is shown in Figure 1. Principal component analysis (PCA) showed two grouped clustering trends with significant overlap ( Figure 1A). A heat map of the top 50 significantly altered metabolites, lipids, and biogenic amines, showed differences in cluster trends between tissue samples at diagnosis and at recurrence ( Figure 1B). There were several significantly upregulated compounds ( Figure 1C) in the lipidomic and biogenic amine analysis. Also included in Figure 1 are scattered column plots for compounds with significant change in abundance at diagnosis and at recurrence ( Figure 1D), which included N-alpha-methylhistamine (P=0.037), 2,3-dihydroxypropyl dihydrogen phosphate with (P=0.029), CE22:6 (P=0.021), LPE 20:4 (P=0.004), LPE 22:6 (P=0.011), LPE 22:4 (P=0.041), CE 20:3 (P=0.031), CE 16:1 (P=0.003), phosphocholine with a (P=0.045), and succinic acid (P=0.025).

**Table 2.** List of significantly altered metabolites in GBM tissue specimens. Comparison of the metabolomic profiles from nine tissue specimens at diagnosis, and three tissue specimens at recurrence. GBM, glioblastoma.

Metabolite name	Super class	Main class	Fold changes	P value
CE 16:1	Sterol Lipids	Sterol esters	2.638	0.003
LPC 20:4	Glycerophospholipids	Glycerophosphocholines	1.8495	0.004
LPE 22:6	Glycerophospholipids	Glycerophosphoethanolamines	3.323	0.011
CE 22:6	Sterol Lipids	Sterol esters	4.5215	0.021
PC 36:4	Sphingolipids	Phosphosphingolipids	1.5981	0.021
FA 22:6 (docosahexaenoic acid)	Fatty Acyls	Fatty acids	1.8499	0.024
succinic acid	TCA acids	TCA acids	2.288	0.025
2,3-Dihydroxypropyl dihydrogen phosphate	Not available	Not available	7.5819	0.029
LPE 20:4	Glycerophospholipids	Glycerophosphoethanolamines	3.9458	0.029
CE 20:3	Sterol Lipids	Sterol esters	2.9844	0.031
glycine	Organic acids	Amino acids and peptides	3.072	0.032
PC 38:3	Glycerophospholipids	Glycerophosphocholines	1.7139	0.035
FA 22:4	Fatty Acyls	Fatty esters	1.9841	0.036
N-alpha-methylhistamine	Organic nitrogen compounds	Amines	8.5724	0.037
LPE 22:4	Glycerophospholipids	Glycerophosphoethanolamines	3.3023	0.041
Phosphocholine	Sphingolipids	Sphingoid bases	2.585	0.045
PC 34:4	Sphingolipids	PHosphosphingolipids	1.5119	0.047

Note: The three tissue specimens at recurrence are paired with three of the nine patients whose tissue specimens were analyzed at diagnosis. Super class and main class were defined in the metabolomics workbench https://www.metabolomicsworkbench.org/databases/refmet/refmet.php.

**Figure 2.**
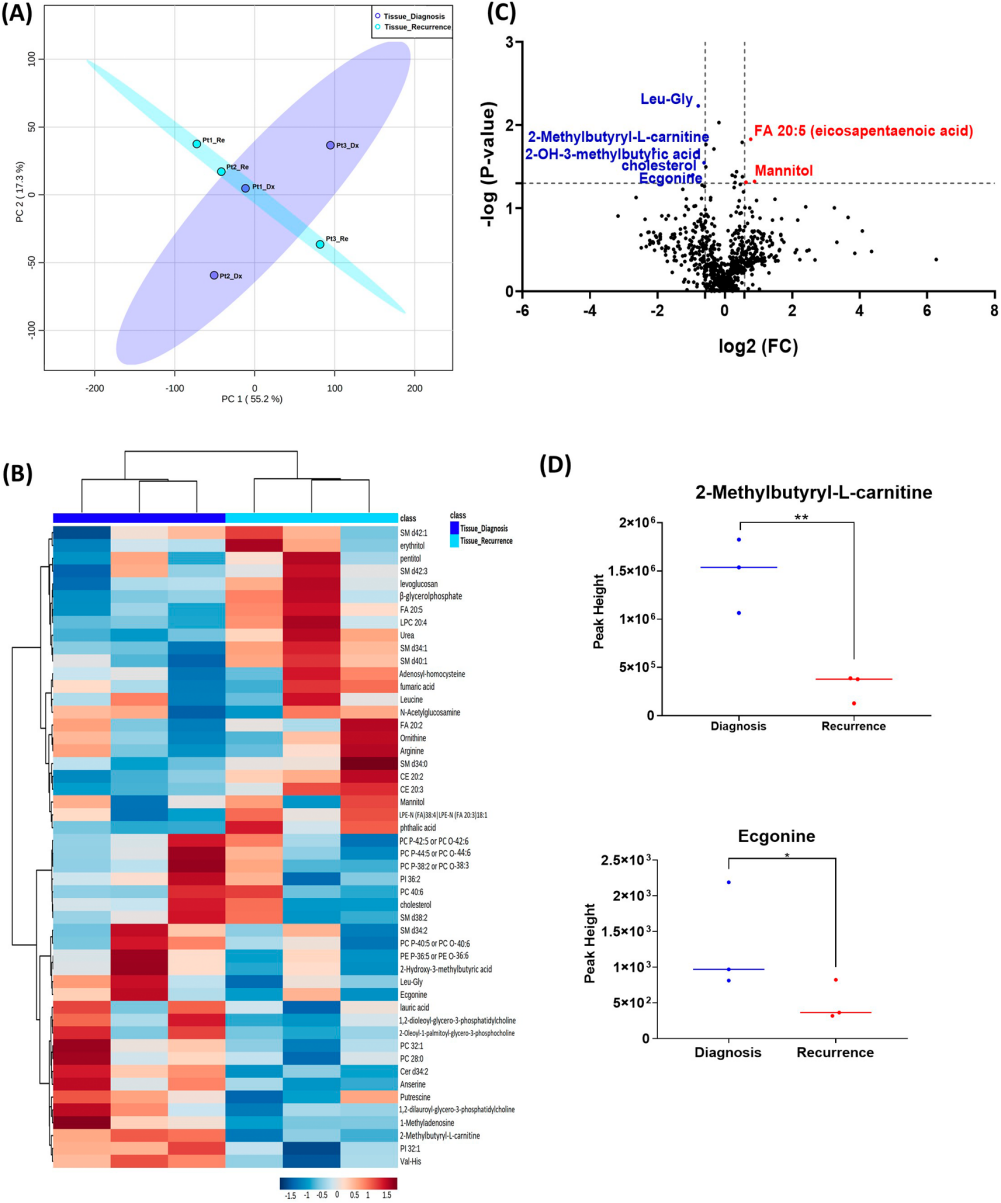
Comparison of metabolomic profiles of three paired fresh frozen brain tumor tissue specimens at diagnosis and at recurrence. (A) Principal component analysis (PCA) plot showing different trends of metabolomic characteristics between tissues at diagnosis (purple) at tissue at recurrence (light blue). (B) Heatmap of the top 50 altered metabolites. Blue indicates decreased peak value and red indicates increased peak value of each compound listed. (C) Volcano plot of upregulated metabolites in red and downregulated metabolites in blue in glioblastoma tumor tissue specimens at recurrence comparing to at diagnosis using p-value of <0.05 and fold change cutoffs of 1.5. (D) Plots of individual values for each metabolite demonstrating peak value changes at diagnosis and at recurrence in brain tumor tissue. Values were determined as peak heights from LC/MS analysis. Single asterisk indicates a p value of <0.05. Double asterisks indicate a p value of <0.01.

When analyzing the three paired tissue specimens at diagnosis and at recurrence, again the PCA plot demonstrated two overlapping clusters ( Figure 2A). The heat map showed more visible separation between tissue samples at diagnosis and at relapse ( Figure 2B). We found 19 compounds that were significantly altered. Volcano plot (using p-value <0.05 and Fold Change cutoff 1.5) revealed that three metabolites were upregulated, and six metabolites were down regulated ( Figure 2C). A complete list of significantly altered metabolites is available in Table 3. Among these metabolites, we found that the levels of the 2-methylbutyryl-L-carnitine (P=0.02) and an unknown compound eluting at 1.84 minutes with an accurate mass of 186.1075 Da (P=0.04) were significantly higher in primary tumors than recurrent GBMs ( Figure 2D).

**Table 3.** List of significantly altered metabolites in three paired GBM tissue specimens at diagnosis and at recurrence. GBM, glioblastoma.

Metabolite name	Super class	Main class	Fold changes *	P value
Leu-Gly	Organic acids	Amino acids and peptides	0.578	0.006
FA 20:5 (eicosapentaenoic acid)	Fatty Acyls	Fatty esters	1.700	0.015
2-Methylbutyryl-L-carnitine	Organic nitrogen compounds	Carnitines	0.231	0.020
2-Hydroxy-3-methylbutyric acid	Fatty Acyls	Fatty acids	0.565	0.021
Cholesterol	Sterol Lipids	Sterols	0.651	0.028
1,2-Dilauroyl-sn-glycero-3-phosphatidylcholine	Not available	Not available	0.516	0.040
mz-rt feature 1.84_186.1075	Alkaloids	Alkaloids	0.478	0.041
Mannitol	Carbohydrates	Monosaccharides	1.840	0.048
levoglucosan	Not available	Not available	1.542	0.049

* For paired analysis, Metaboanalyst calculates fold changes (FC) by computing the ratio between paired samples (i.e., one FC per pair), and then computing their means (i.e., pair means). Super class and main class were defined in the metabolomics workbench https://www.metabolomicsworkbench.org/databases/refmet/refmet.php.

### Unsupervised exploratory analysis on plasma metabolomic, biogenic amine, and lipidomic characteristics

**Figure 3.**
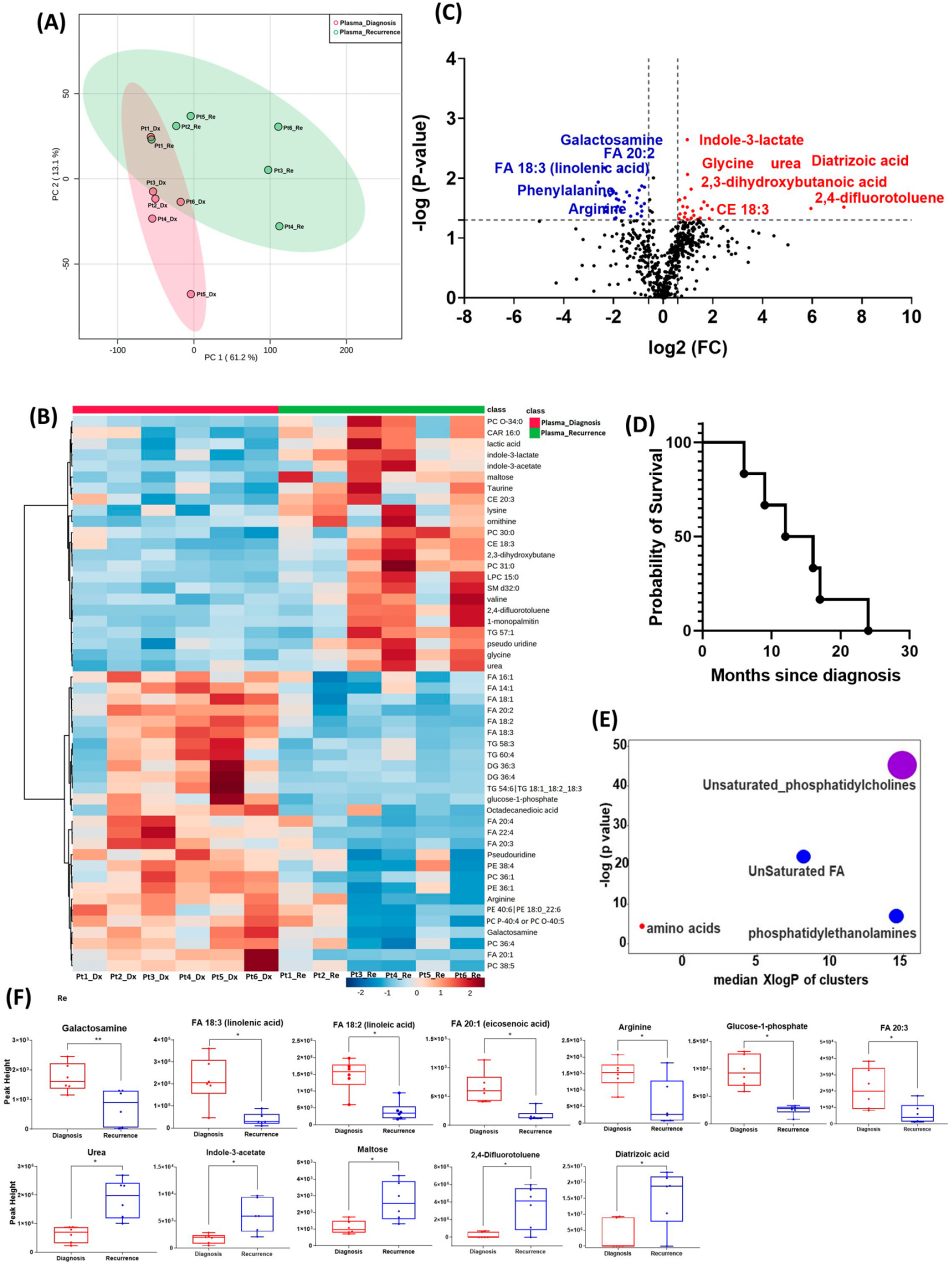
Comparison of metabolomic profiles in plasma of glioblastoma patients at diagnosis and at recurrence. (A) Principal component analysis (PCA) plot showing different trends of metabolomic characteristics between tissues at diagnosis (purple) at tissue at recurrence (light blue). (B) Heatmap of the top 50 altered metabolites. Blue indicates decreased peak value and red indicates increased peak value of each compound listed. (C) Volcano plot of up regulated metabolites in red and down regulated metabolites in blue in glioblastoma tumor tissue specimens at recurrence comparing to at diagnosis using p-value of <0.05 and fold change cutoffs of 1.5. Single asterisk indicates a p value of <0.05. Double asterisks indicate a p value of <0.01. (D) Progression free survival of six patients whose plasma samples were analyzed. (E) Significantly altered metabolite clusters by ChemRich, a statistical enrichment approach based on chemical similarity. The size of the dots is in proportion with the level of alteration in each cluster of metabolites. Red indicates increased peak value and blue indicates decreased peak value. (F) Box plots demonstrating metabolite peak value changes at diagnosis and at recurrence in brain tumor tissues. Values were determined as peak height from LC/MC analysis. Single asterisk indicates a p value of <0.05. Double asterisks indicate a p value of <0.01.

All six patients enrolled in this cohort had paired plasma specimens at diagnosis and at recurrence. However, these were not the same patients whose GBM tissue were studied in the cohort above. PCA analysis showed evident separation between the metabolomic profiles at diagnosis and at recurrence, except for patient one, whose metabolomic profiles were similar at diagnosis and at recurrence ( Figure 3A). The heatmap of the top 50 altered metabolites showed visible differences between plasma at diagnosis and at recurrence ( Figure 3B). Again, the metabolite profile of patient one at recurrence was similar to its status at diagnosis, while the other patients’ profiles demonstrated differences.

**Figure 4.**
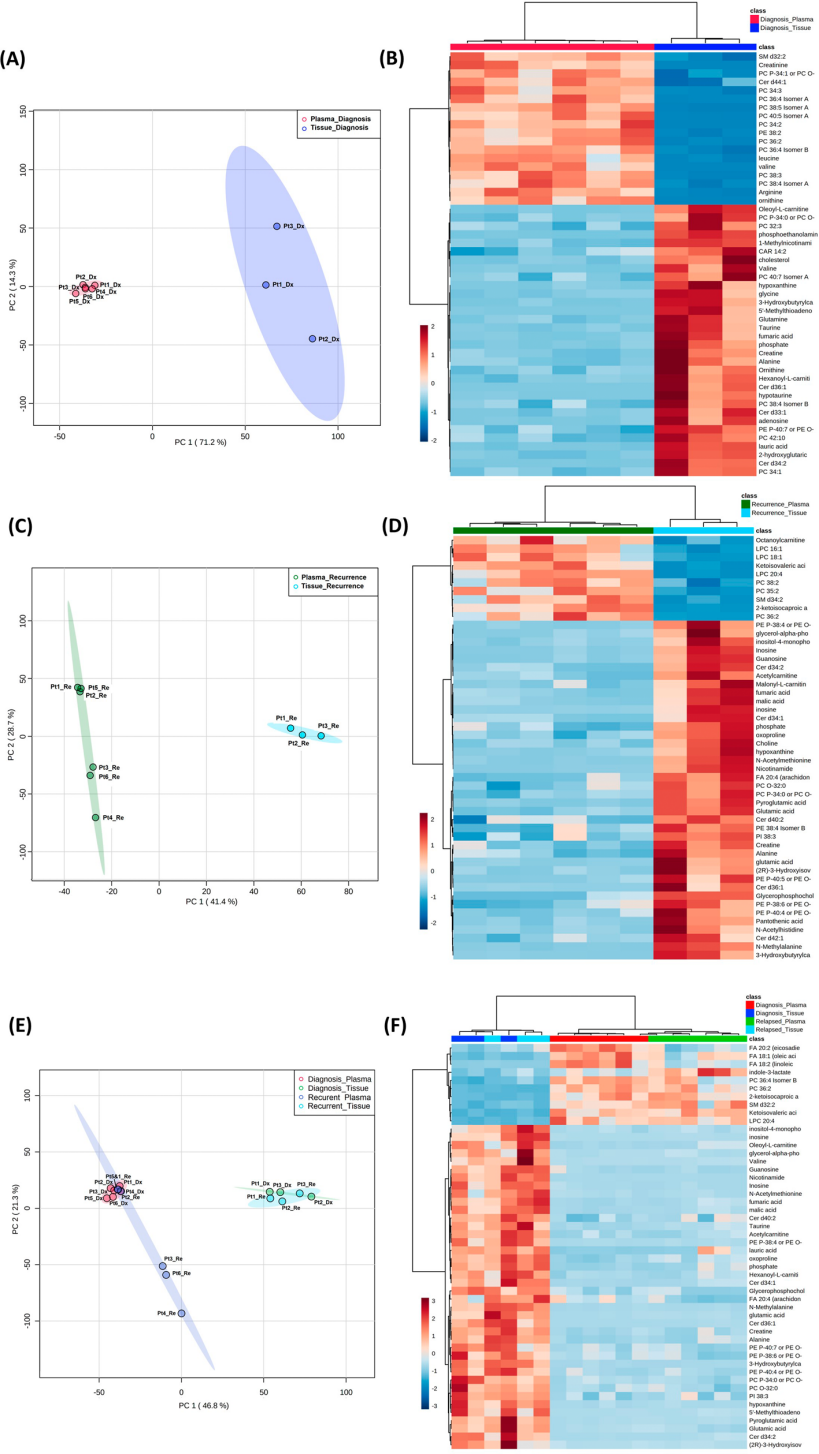
Comparison of metabolomic profiles in glioblastoma tumor tissue and patient plasma. (A, C, E) Principal component analysis plots comparing tissue *vs.* plasma specimens at diagnosis, tissue *vs.* plasma at recurrence, and all four specimen groups. (B, D, F) Heatmaps of the top 50 significantly altered metabolites in the comparisons correlating with A, C, and E, respectively.

There were 61 compounds that were altered significantly between diagnosis and recurrence in the patient plasma. The representatives were shown in Figure 3C. A complete list of significantly altered metabolites is available in Table 4. The progression free survival of these six patients was shown in Figure 3D. Using ChemRich, we were able to identify that based on chemical structural similarity, amino acids and unsaturated phosphatidylcholines were significantly up regulated and unsaturated fatty acids and phosphatidylethanolamines were downregulated ( Figure 4E). The compounds with significantly increased abundance at recurrence included 2,4-difluorotoluene (P=0.031), diatrizoic acid (P=0.032), indole-3-acetate with (P=0.029), urea (P=0.025), pseudo uridine (P=0.042), and maltose (P=0.035). The compounds with significantly decreased abundance included FA 20:1 (eicosenoic acid) (P=0.017), glucose-1-phosphate (P=0.017), FA 18:2 (linoleic acid) (P=0.017), arginine (P=0.036), FA 20:3 (homo-gamma-linolenic acid) (P=0.036), galactosamine (P=0.007), and FA 18:3 (linolenic acid) (P=0.012) ( Figure 3F).

**Table 4.** List of significantly altered metabolites in paired GBM patient plasma specimens at diagnosis and at recurrence. GBM, glioblastoma.

Metabolite name	Super class	Main class	Fold changes *	P value
Indole-3-lactate	Organoheterocyclic compounds	Indoles	1.9588	0.002
Galactosamine	Carbohydrates	Monosaccharides	0.1962	0.007
FA 20:2 (eicosadienoic acid)	Fatty Acyls	Fatty esters	0.31436	0.007
glycine	Organic acid	Amino acids and peptides	1.9691	0.009
PC 40:4	Sphingolipids	Phosphosphingolipids	0.76047	0.010
FA 18:3 (linolenic acid)	Fatty Acyls	Fatty esters	0.16342	0.012
PC 36:1	Sphingolipids	Phosphosphingolipids	0.55077	0.014
PC 36:4 Isomer B	Glycerophospholipids	Glycerophosphocholines	0.59434	0.014
2,3-dihydroxybutanoic acid	Carbohydrates	Monosaccharides	2.1832	0.015
FA 18:2 (linoleic acid)	Fatty Acyls	Fatty esters	0.23898	0.015
FA 14:1 (physeteric acid)	Fatty Acyls	Fatty esters	0.51145	0.016
FA 20:1 (eicosenoic acid)	Fatty Acyls	Fatty esters	0.24899	0.017
PE 36:1	Glycerophospholipids	Glycerophosphoethanolamines	0.36148	0.017
glucose-1-phosphate	Carbohydrates	Monosaccharides	0.24717	0.017
PC 34:2	Sphingolipids	Phosphosphingolipids	0.26846	0.018
FA 22:4	Fatty Acyls	Fatty esters	0.2265	0.019
Phenylalanine	Organic acids	Amino acids and peptides	0.19823	0.019
PE 40:6|PE 18:0_22:6	Glycerophospholipids	Glycerophosphoethanolamines	0.38988	0.020
beta-alanine	Organic acids	Amino acids and peptides	1.8339	0.021
Glutamic acid	Organic acids	Amino acids and peptides	0.48566	0.021
PC 40:5 Isomer B	Glycerophospholipids	Glycerophosphocholines	0.27183	0.022
TG 50:0	Glycerolipids	Triradylglycerols	0.68725	0.023
isocitric acid	Organic acids	TCA acids	1.5782	0.023
PC 40:5 Isomer A	Sphingolipids	Phosphosphingolipids	0.2885	0.023
urea	Organic acids	Carboximidic acids	3.1119	0.025
TG 58:3	Glycerolipids	Triradylglycerols	0.33746	0.025
FA 18:1 (oleic acid)	Sphingolipids	Ceramides	0.47341	0.026
3-Hydroxyvaleric acid	Fatty Acyls	Fatty acids	0.59139	0.027
indole-3-acetate	Organoheterocyclic compounds	Indolyl carboxylic acids	3.4338	0.029
PE 38:6	Glycerophospholipids	Glycerophosphoethanolamines	0.54073	0.029
glutaric acid	Fatty Acyls	Fatty acids	1.7297	0.030
Cer 43:1	Sphingolipids	Ceramides	0.28123	0.030
TG 58:0	Glycerolipids	Triradylglycerols	1.9617	0.030
PC 38:5 Isomer A	Glycerophospholipids	Glycerophosphocholines	0.20248	0.031
2,4-difluorotoluene	Not available	Not available	154.28	0.031
Diatrizoic acid	Not available	Not available	61.625	0.032
SM d32:0	Sphingolipids	Sphingomyelins	2.8897	0.032
CE 18:3	Sterol Lipids	Sterol esters	3.9288	0.033
FA 22:6 (docosahexaenoic acid)	Fatty Acyls	Fatty esters	0.31099	0.034
TG 60:4	Glycerolipids	Triradylglycerols	0.26613	0.035
PC 36:2	Glycerophospholipids	Glycerophosphocholines	0.69347	0.035
maltose	Carbohydrates	Disaccharides	2.2858	0.035
PE 38:2	Glycerophospholipids	Glycerophosphoethanolamines	0.5522	0.035
Arginine	Organic acids	Amino acids and peptides	0.20443	0.036
FA 20:3 (homo-gamma-linolenic acid)	Fatty Acyls	Fatty esters	0.20424	0.036
FA 14:0 (myristic acid)	Fatty Acyls	Fatty esters	0.73643	0.037
PC P-42:3 or PC O-42:4	Glycerophospholipids	Glycerophosphocholines	1.8478	0.038
FA 16:1 (palmitoleic acid)	Fatty Acyls	Fatty esters	0.48553	0.039
PC P-40:5 or PC O-40:6	Glycerophospholipids	Glycerophosphocholines	1.5978	0.040
pseudo uridine	Nucleic acids	Pyrimidines	2.3559	0.042
PC 38:6	Glycerophospholipids	Glycerophosphocholines	0.5424	0.043
DG 36:3	Glycerolipids	Glycosyldiradylglycerols	0.40467	0.043
valine	Organic acids	Amino acids and peptides	1.8064	0.045
DG 36:4 Isomer A	Glycerolipids	Glycosyldiradylglycerols	0.26795	0.046
TG 46:4 Isomer A	Glycerolipids	Triradylglycerols	2.7944	0.046
1-monopalmitin	Not available	Not available	2.186	0.047
PC P-40:4 or PC O-40:5	Glycerophospholipids	Glycerophosphocholines	0.25861	0.048
LPC 15:0	Glycerophospholipids	Glycerophosphocholines	3.6157	0.048
LPC 20:4.1	Glycerophospholipids	Glycerophosphocholines	1.5535	0.048
CE 18:0	Sterol Lipids	Sterol esters	2.6094	0.049
Taurine	Organic acids	Sulfonic acids	1.9581	0.049

* For paired analysis, Metaboanalyst calculates fold changes (FC) by computing the ratio between paired samples (i.e., one FC per pair), and then computing their means (i.e., pair means). Super class and main class were defined in the metabolomics workbench https://www.metabolomicsworkbench.org/databases/refmet/refmet.php.

### Combined analyses on glioblastoma tissue and patient plasma at diagnosis and at recurrence

Both the PCA and heatmap analyses showed separate metabolic profiles from tissues at diagnosis and plasma at diagnosis ( Figure 4A and B). Similarly, the metabolomic profile from tissue at recurrence separated from plasma at recurrence ( Figure 4C and D). When placing all four groups of data in one plot ( Figure 4C and D), we again saw inter-specimen differences between the metabolomic profiles in tissue and plasma, but there were no intra-specimen differences at diagnosis and at recurrence.

## Discussion

In this study, we investigated the comprehensive untargeted metabolomic, lipidomic, and biogenic amine profiles of GBM tissue and patient plasma specimens at diagnosis and at recurrence. Despite a small overall cohort size, our result showed that many metabolites were altered in GBM tissue and patient plasma at recurrence when compared to diagnosis. Our study demonstrated the feasibility of studying GBM tissue and patient blood specimen longitudinally using metabolomic methodology.

GBM display marked metabolic heterogeneity in their microenvironments. ^17^ Both glucose and lipid metabolisms are abnormally regulated in GBM tissues. ^18^^,^^19^ In our study, we observed several metabolites that had changed in abundance at recurrence when compared to diagnosis. Many of these metabolites were also identified in a recently published study on the metabolic hallmarks of gliomas. ^20^ Specifically, we identified 2-methylbutyryl-L-carnitine that was known to reflect tumor metabolic flexibility in brain tumor tissues at diagnosis and at recurrence. Carnitine serves as a “shuttle-molecule” that allows fatty acid acyl moieties to enter the mitochondrial matrix for oxidization via the beta-oxidation pathway. ^21^ We found that the 2-methylbutyryl-L-carnitine level was significantly reduced in recurrent tumors compared to initial GBM tissue. Carnitine transporter modulation has been thought to be a potential target for cancer treatment. ^21^ In addition, we also found many altered levels of lipids in GBM tissue at recurrence when compared to initial diagnosis. Our findings are in line with previously published data suggesting lipid metabolic alterations in GBM. ^22^ In addition, mannitol was upregulated in recurrent tissue compared to the original tumor, suggesting BBB permeability changes after surgical resection and chemoradiation. This may suggest mannitol as a vehicle to guide targeted treatment.

Notably, the list of metabolites with significantly altered abundance and fold changes differ when comparing the unpaired samples (nine tissue samples at diagnosis *vs.* three tissue samples at recurrence) and the paired samples (three tissue samples from the same patients at diagnosis and at recurrence). Despite a small sample size, the paired tissues samples demonstrated a clearer trend in the differences in compound abundance at diagnosis and at recurrence ( Figure 1A compared with Figure 2A). It is likely that the effect size was reduced because of the heterogeneity of unpaired samples. Based on these findings, we recommend that future studies utilize paired samples due to their superior ability to serve as an internal control. The paired samples offer a more reliable basis for assessing changes in metabolite abundance between diagnosis and recurrence, providing greater confidence in the observed differences.

In both the PCA plot from patient plasma and the heatmap generated from the top 50 altered metabolites, we found that the metabolomic profiles differed between specimens at diagnosis and at recurrence, except for one patient with early recurrence. The metabolomic profile of this patient’s plasma at diagnosis was different from the rest of the plasma specimens at diagnosis and was similar to the group pattern at recurrence. This notable finding needs to be validated in a larger cohort of patients with treatment refractory tumors and early recurrence. This metabolomic signature may indicate a high risk and poor prognosis.

In the plasma cohort, we found several significantly altered metabolites with large fold changes. For example, 2,4-difluorotoluene increased in patient plasma at recurrence; this metabolite is incorporated into DNA and undergoes replication by DNA polymerase enzymes. ^23^ The observed change may suggest rapid growth of recurrent tumors. However, it is worth noting that this compound is also used as an intermediate in the production of pharmaceuticals and dyes. Therefore, it is crucial to validate this finding through further investigation in future studies. Diatrizoic acid also increased at recurrence. This compound is a contrast agent used during imaging and can be attributed to a previous injection for neuroimaging prior to blood sample collection. Indole-3-acetate is an indol-3-yl carboxylic acid anion and has a role as a human metabolite. We again found several other compounds involved in glucose and lipid metabolism. The overall pattern changes of these compounds need to be validated in targeted metabolomics for their potential candidacy as biomarkers for treatment response and tumor recurrence. The enrichment of these metabolites in recurrent GBM tissue may suggest that targeting metabolic activity can be a potential adjuvant targeted treatment for patients with GBM.

Of note, when plotting all four groups in the same PCA plot, the brain tissue and plasma specimens separated distinctly from each other regardless of disease status ( Figure 4). There was no overlap between specimen types, while the intra-specimen comparison at diagnosis and at recurrence became smaller. This suggested that plasma metabolite patterns are not reflective of brain tumor tissue, and therefore, two sets of biomarker panels are necessary for tissue and plasma. The pattern of tumor microenvironment can be further altered through end organ metabolism in the plasma. Also, plasma is affected by systemically administered medication for peri-operative care.

Our study has limitations. First, we must acknowledge that the sample size in this pilot study is small. Second, although we have paired data within the brain tissue and plasma cohorts at diagnosis and at recurrence, we were unable to identify if the tissue and plasma specimens were from the same patients due to the restrictions of the UC Davis biorepository consents for GBM tissue. In addition, we did not have normal brain tissue or plasma for comparison for ethical reasons. We also recognized that the blood metabolomic profiles of healthy volunteers vary ^24^^–^^26^ and the metabolic signatures of normal controls can also indicate early states of certain diseases, ^27^ drug effects, ^24^ and dietary composition. ^28^ We will obtain age, sex, and ethnicity matched controls in the future study to minimize potential confounding effects. These limitations prevented us from identifying small changes in metabolite abundance. Therefore, only significant and large fold changes were analyzed. A bigger cohort with paired specimens will be emphasized in future studies. The current study paved the way for the next targeted metabolomics, lipidomic, and biogenic amine studies validating and further investigating these profiles.

## Conclusions

Our data suggest that metabolomic profiles of human GBM tissue and patient plasma differ at diagnosis and at recurrence. Many metabolites involved in tumorigenesis and metabolomic flexibility were identified. A larger study using target metabolomic assay is warranted to measure the levels of these metabolites, which will help identify the metabolomic signatures in both GBM tissue and patient plasma for risk stratification, clinical outcome prediction, and development of new adjuvant metabolomic-targeting therapy.

## Author contributions

All authors contributed to the study conception and design. Material preparation, data collection and analysis were performed by Yin Allison Liu, Orwa Aboud, Lina Dahabiyeh, Orin Bloch, and Oliver Fiehn. The first draft of the manuscript was written by Yin Allison Liu, and all authors commented on previous versions of the manuscript. All authors read and approved the final manuscript.

## Data Availability

Figshare: Brain tissue Biogenic Amines 10.9.22.xlsx/. https://doi.org/10.6084/m9.figshare.24769668.v2. ^29^ Data are available under the terms of the Creative Commons Attribution 4.0 International license (CC-BY 4.0).
