# Supplementary material for: Metabolomic characterization of human glioblastomas and patient plasma: a pilot study
Source: F1000Res. 2024 Sep 19;13:98. Originally published 2024 Feb 16. [Version 5] doi: 10.12688/f1000research.143642.5 (PMC11452765; doi:10.12688/f1000research.143642.5)
Supplement: Peer review history

# Reviewer report: Reviewer response for version 5

**DOI:** 10.5256/f1000research.172098.r325276

**Published:** 2024-10-04

**Authors:** Andrew J Scott

**Affiliations:** 1 University of Michigan, Ann Arbor, USA

The authors have satisfactorily amended the references of concern.

Is the work clearly and accurately presented and does it cite the current literature?

Partly

If applicable, is the statistical analysis and its interpretation appropriate?

Partly

Are all the source data underlying the results available to ensure full reproducibility?

Yes

Is the study design appropriate and is the work technically sound?

Partly

Are the conclusions drawn adequately supported by the results?

No

Are sufficient details of methods and analysis provided to allow replication by others?

Partly

Reviewer Expertise:

glioma metabolism, metabolomics

I confirm that I have read this submission and believe that I have an appropriate level of expertise to confirm that it is of an acceptable scientific standard.

# Reviewer report: Reviewer response for version 4

**DOI:** 10.5256/f1000research.171030.r319005

**Published:** 2024-09-17

**Authors:** Andrew J Scott

**Affiliations:** 1 University of Michigan, Ann Arbor, USA

The sentence of concern is unfortunately still not correctly cited. "These studies have identified unique metabolic signatures associated with survival,(14) immune tolerance of GBM,(15) and metabolic features indicative of accelerated anabolic metabolism.(9)" Reference 9 is a phase 2 trial of TMZ/RT - I believe the authors meant to cite the paper that is currently reference #12 by Chinnaiyan et al.

Is the work clearly and accurately presented and does it cite the current literature?

Partly

If applicable, is the statistical analysis and its interpretation appropriate?

Partly

Are all the source data underlying the results available to ensure full reproducibility?

Yes

Is the study design appropriate and is the work technically sound?

Partly

Are the conclusions drawn adequately supported by the results?

No

Are sufficient details of methods and analysis provided to allow replication by others?

Partly

Reviewer Expertise:

glioma metabolism, metabolomics

I confirm that I have read this submission and believe that I have an appropriate level of expertise to confirm that it is of an acceptable scientific standard, however I have significant reservations, as outlined above.

# Reviewer report: Reviewer response for version 3

**DOI:** 10.5256/f1000research.169444.r306173

**Published:** 2024-08-23

**Authors:** Andrew J Scott

**Affiliations:** 1 University of Michigan, Ann Arbor, USA

The authors have addressed some of the previous comments. However, neither reference (a) nor (c) from previous comment 3 have been added. These should be addressed as they are large and more extensive metabolomics studies of GBM in human and preclinical models. In particular, the sentence "These studies have identified unique metabolic signatures associated with survival,(7) immune tolerance of GBM(8), and metabolic features indicative of accelerated anabolic metabolism(9)" in the introduction is mis-referenced and should be amended with the references listed previously.

Is the work clearly and accurately presented and does it cite the current literature?

Partly

If applicable, is the statistical analysis and its interpretation appropriate?

Partly

Are all the source data underlying the results available to ensure full reproducibility?

Yes

Is the study design appropriate and is the work technically sound?

Partly

Are the conclusions drawn adequately supported by the results?

No

Are sufficient details of methods and analysis provided to allow replication by others?

Partly

Reviewer Expertise:

glioma metabolism, metabolomics

I confirm that I have read this submission and believe that I have an appropriate level of expertise to confirm that it is of an acceptable scientific standard, however I have significant reservations, as outlined above.

# Response

**Published:** 2024-08-26

**Authors:** Yin Allison Liu

**Affiliations:** Neurology and Neurosurgery, University of California Davis Health System, Sacramento, California, USA

We apologize for the miscitations. These have been amended in the new version.

# Reviewer report: Reviewer response for version 2

**DOI:** 10.5256/f1000research.168028.r295614

**Published:** 2024-07-18

**Authors:** Joseph Chen

**Affiliations:** 1 University of Louisville, Louisville, Kentucky, USA

The authors have addressed the concerns raised, and the updated report is improved and appropriately recognizes and discusses the limitations of this study. Minor changes are recommended.

 Table 1 should be updated to more clearly define the paired tissue details. It’s a bit confusing since the paired title is directly above the values, whereas the complete GBM cohort is to the left of the values. It may be better to have the cohort information above the “clinical and molecular features” and “demographic profiles” headings. Additionally, I would recommend providing a small break in the table above the plasma data sets for clarity.

Is the work clearly and accurately presented and does it cite the current literature?

Partly

If applicable, is the statistical analysis and its interpretation appropriate?

Yes

Are all the source data underlying the results available to ensure full reproducibility?

Yes

Is the study design appropriate and is the work technically sound?

Partly

Are the conclusions drawn adequately supported by the results?

Partly

Are sufficient details of methods and analysis provided to allow replication by others?

Yes

Reviewer Expertise:

GBM tumor microenvironment, biomaterials, mechanobiology, proteomics, extracellular matrix, cell migration

I confirm that I have read this submission and believe that I have an appropriate level of expertise to confirm that it is of an acceptable scientific standard.

# Response

**Published:** 2024-07-19

**Authors:** Yin Allison Liu

**Affiliations:** Neurology and Neurosurgery, University of California Davis Health System, Sacramento, California, USA

Thank you for your comments. We have edited the table according to your recommendations.

# Reviewer report: Reviewer response for version 1

**DOI:** 10.5256/f1000research.157324.r260753

**Published:** 2024-05-23

**Authors:** Andrew J Scott

**Affiliations:** 1 University of Michigan, Ann Arbor, USA

Glioblastoma (GBM) is one of the most difficult to treat cancers, and the investigation of metabolites in patients may represent a potential strategy to develop new stratification, prediction and treatment modalities. In this study, a small panel of tumor tissue and plasma samples from GBM patients are subjected to metabolomic analysis in an effort to determine if metabolites differ between primary and recurrent GBM. Plasma samples are also investigated, although these samples were from a different cohort of patients. Several differences are noted in select metabolites, although sample size is small (n=3 recurrent tumors compared with n=3 matched primary tumors, or n=3 recurrent tumors compared to the 3 matched primary + additional unmatched samples). For this reason the conclusions regarding metabolite differences between primary and recurrent GBM tumors may not be reliable. Moreover, further details regarding methodological and analytical descriptions are needed. Concerns are described below.

 1) Acquisition of patient-matched primary and recurrent tumors is important for determining new prognostic/therapeutic approaches and is a strength of the study, but sample number is a major concern. It is difficult to draw reliable conclusions and interpretations from an n=3 group of patients, in particular with regard to conclusions about changes in individual metabolite levels between primary and recurrent tumors. How reliable are these findings when false discovery rates are considered?

 2) While analyses of individual metabolites (e.g., Figs 1D, 2D, 3B) lack sufficient statistical power, pathway-level (rather than metabolite-level) analysis tools are available in MetaboAnalyst that incorporate comprehensive metabolomic data (such as pathway analysis or MSEA, somewhat analogous to GSEA of geneset data) to generate metabolite enrichment and pathway predictions. These tools may be useful to more reliably and repeatably inform about metabolic states of tumors.

 3) The authors understate the current literature on GBM metabolomic profiling and imply this has not been done before. Several larger scale studies have already performed metabolomic profiling of GBMs and other high-grade gliomas and identified unique metabolic signatures, including (unmatched) primary vs. recurrent analyses. These datasets included stratification into various subgroups and correlations with patient medical and outcome data. Relevant DOIs are: 

 a.) Scott AJ . et.al., 2023

 b.) Chinnaiyan P. et.al., 2012

 c.) Kesarwani P .et.al., 2019

 Further expansion into the literature and contextualization of the authors' findings with these existing data are necessary.

 4) In Table 1, further clarification on which patients were used in the different matched vs. unmatched analyses is needed.

 5) Throughout the text and figures, the authors use phrases such as “top 50 altered metabolites,” “significantly altered/different abundance/fold change,” etc. These are vague and statistically undefined, unclear.

**Minor comments**

 6) Description of clustering methods/algorithms used to produce dendrograms for the heatmaps were not found.

 7) More contrast or use of color-blind palettes would assist readability of figure panels (Figs 1A, 1B, 2A, 2B, 4C, 4D).

Is the work clearly and accurately presented and does it cite the current literature?

Partly

If applicable, is the statistical analysis and its interpretation appropriate?

Partly

Are all the source data underlying the results available to ensure full reproducibility?

Yes

Is the study design appropriate and is the work technically sound?

Partly

Are the conclusions drawn adequately supported by the results?

No

Are sufficient details of methods and analysis provided to allow replication by others?

Partly

Reviewer Expertise:

Cancer and brain metabolism, metabolomics, therapy resistance

I confirm that I have read this submission and believe that I have an appropriate level of expertise to confirm that it is of an acceptable scientific standard, however I have significant reservations, as outlined above.

## References

[1] : Metabolomic Profiles of Human Glioma Inform Patient Survival. *Antioxid Redox Signal* .2023;39(13-15) : 10.1089/ars.2022.0085 942-956 10.1089/ars.2022.0085 36852494 PMC10655010

[2] : The metabolomic signature of malignant glioma reflects accelerated anabolic metabolism. *Cancer Res* .2012;72(22) : 10.1158/0008-5472.CAN-12-1572-T 5878-88 10.1158/0008-5472.CAN-12-1572-T 23026133 PMC4638418

[3] : Metabolic remodeling contributes towards an immune-suppressive phenotype in glioblastoma. *Cancer Immunol Immunother* .2019;68(7) : 10.1007/s00262-019-02347-3 1107-1120 10.1007/s00262-019-02347-3 31119318 PMC6586493

# Response

**Published:** 2024-06-16

**Authors:** Yin Allison Liu

**Affiliations:** Neurology and Neurosurgery, University of California Davis Health System, Sacramento, California, USA

1) Acquisition of patient-matched primary and recurrent tumors is important for determining new prognostic/therapeutic approaches and is a strength of the study, but sample number is a major concern. It is difficult to draw reliable conclusions and interpretations from an n=3 group of patients, in particular with regard to conclusions about changes in individual metabolite levels between primary and recurrent tumors. How reliable are these findings when false discovery rates are considered?

**Response: **Thank you for the feedback. We acknowledge that the small sample size may compromise the reliability of our conclusions, making it challenging to draw definitive results. This study serves as a pilot based on the availability of specimens in the biobank, and we intend to use these preliminary findings to support our application for a larger grant to fund expanded biospecimen collection. Following FDR batch correction, we found no significant compounds, which we must acknowledge. We have submitted grant proposals with the intention that, if funded, we will have access to a substantial collection of specimens and plasma for comprehensive analysis.

 2) While analyses of individual metabolites (e.g., Figs 1D, 2D, 3B) lack sufficient statistical power, pathway-level (rather than metabolite-level) analysis tools are available in MetaboAnalyst that incorporate comprehensive metabolomic data (such as pathway analysis or MSEA, somewhat analogous to GSEA of geneset data) to generate metabolite enrichment and pathway predictions. These tools may be useful to more reliably and repeatably inform about metabolic states of tumors.

**Response: **We used ChemRICH ( ChemRICH (ucdavis.edu)), a tool developed in our core lab and a statistical enrichment approach based on chemical similarity to classify our data, and the results are shown in Figure 3(E). We did not perform this enrichment analysis for the tissues demonstrated in Figures 1 and 2 due to the very small sample size.

 3) The authors understate the current literature on GBM metabolomic profiling and imply this has not been done before. Several larger scale studies have already performed metabolomic profiling of GBMs and other high-grade gliomas and identified unique metabolic signatures, including (unmatched) primary vs. recurrent analyses. These datasets included stratification into various subgroups and correlations with patient medical and outcome data. Relevant DOIs are: 

 a.) Scott AJ . et.al., 2023

 b.) Chinnaiyan P. et.al., 2012

 c.) Kesarwani P .et.al., 2019

 Further expansion into the literature and contextualization of the authors' findings with these existing data are necessary.

**Response: **Thank you for the suggested references. We have expanded our discussion and added the references.

 4) In Table 1, further clarification on which patients were used in the different matched vs. unmatched analyses is needed.

**Response: **We have added a column to indicate the matched tissue specimens and also included notation on whether the specimens are paired or unpaired for each specimen type.

 5) Throughout the text and figures, the authors use phrases such as “top 50 altered metabolites,” “significantly altered/different abundance/fold change,” etc. These are vague and statistically undefined, unclear.

**Response:** “Top 50 altered" denotes the top 50 metabolites with significantly altered abundance in each comparison, exhibiting fold changes greater than 1.5. This includes both metabolites with increased and decreased fold changes. We have added this in the result section.

**Minor comments**

 6) Description of clustering methods/algorithms used to produce dendrograms for the heatmaps were not found.

**Response:** Dendrogram and heat map were produced from MetaboAnalyst version 4.0 using the default Euclidean distance and Ward linkage. We have added this in the result section.

 7) More contrast or use of color-blind palettes would assist readability of figure panels (Figs 1A, 1B, 2A, 2B, 4C, 4D).

**Response: **We have regenerated the figures with improved contrast resolution. Additionally, we have selected colors that are friendly for individuals with color blindness.

# Reviewer report: Reviewer response for version 1

**DOI:** 10.5256/f1000research.157324.r251328

**Published:** 2024-03-20

**Authors:** Joseph Chen

**Affiliations:** 1 University of Louisville, Louisville, Kentucky, USA

COMMENTS TO AUTHORS

 In this report, the authors present a pilot study to explore the utility of metabolomic characterization as a tool for risk stratification, outcome prediction, and the identification of new metabolic-targeting therapies. The authors use untargeted assays to analyze metabolomic, lipidomic, and biogenic amine profiles of GBM tissue and plasma from a mixed cohort of 15 patients. The investigation revealed distinct metabolic, lipidomic, and biogenic amine profiles between primary and recurrent states in both tissue and plasma. Additionally, the data revealed that metabolomic profiles also differed from tissue and plasma samples. This study identifies several metabolites in the tissue that corroborate findings in the literature; however, the plasma data appears to identify secondary metabolites from peri-operative care that may confound the interpretation. Although this report indicates the possibility of leveraging metabolomic tools to inform patient prognosis, this study has many limitations that hamper these conclusions. These include includes the small sample size, the lack of controls, and the inability to directly analyze tissue and plasma from the same patient. Additionally, the clearer separation in metabolite data in the patient matched tissue cohort (n=3) highlights the limitation of their bulk/combined analysis and reduces the confidence in their results. Given these limitations, it is unclear whether there is sufficient data to support the author’s conclusions.

 MAJOR COMMENTS:

1. Details on the patient demographics in the GBM tissue cohort is limited. Why are there no details on IDH, EGFR, or MGMT status in the patient tissue? Which three patients were part of the matched groups? Where is the prognosis data for this group? With such small sample sizes, the addition of patient data is needed and may identify new insights and interpretations.
2. With the list of altered metabolites being different (Table 2 and Table 3), how do the authors parse out the relevant metabolic alterations that occur during recurrence? Is the bulk primary tumor comparison with 3 recurrent tumors useful or does it confound the patient-matched analysis?
3. Interpretation of the 1 plasma sample that showed similar metabolic profiles is premature. The authors’ conclusion that this sample may be evidence of an aggressive signature seems unwarranted as it would suggest that no metabolic alterations at the plasma level leads to early recurrence and patient death. Alternatively, the data may point to a temporal effect of plasma analysis, where in early recurrence, only a short time has elapsed and thus plasma levels are more similar. If this were true, additional analysis would be needed to detail the duration of time between measurements and may suggest strong variance in plasma level analysis.

MINOR COMMENTS:

1. There are several reports within recent years that have investigated the metabolome in GBM. A more exhaustive survey of the literature is needed.
2. Figure 1D lists N-alpha-methylhistamine twice

Is the work clearly and accurately presented and does it cite the current literature?

Partly

If applicable, is the statistical analysis and its interpretation appropriate?

Yes

Are all the source data underlying the results available to ensure full reproducibility?

Yes

Is the study design appropriate and is the work technically sound?

Partly

Are the conclusions drawn adequately supported by the results?

Partly

Are sufficient details of methods and analysis provided to allow replication by others?

Yes

Reviewer Expertise:

GBM tumor microenvironment, biomaterials, mechanobiology, proteomics, extracellular matrix, cell migration

I confirm that I have read this submission and believe that I have an appropriate level of expertise to confirm that it is of an acceptable scientific standard, however I have significant reservations, as outlined above.

# Response

**Published:** 2024-06-16

**Authors:** Yin Allison Liu

**Affiliations:** Neurology and Neurosurgery, University of California Davis Health System, Sacramento, California, USA

MAJOR COMMENTS:

1. Details on the patient demographics in the GBM tissue cohort is limited. Why are there no details on IDH, EGFR, or MGMT status in the patient tissue? Which three patients were part of the matched groups? Where is the prognosis data for this group? With such small sample sizes, the addition of patient data is needed and may identify new insights and interpretations.

**Response: **The current biobank has an IRB protocol that limits access to information beyond general tumor pathology diagnosis, age at diagnosis, sex, and ethnicity. We have included the details of matched patients in Table 1. Our aim is to use this study as a basis for justifying a grant that would enable the establishment of a biobank with comprehensive clinical information. This initiative reflects a valuable lesson learned by our team.

1. With the list of altered metabolites being different (Table 2 and Table 3), how do the authors parse out the relevant metabolic alterations that occur during recurrence? Is the bulk primary tumor comparison with 3 recurrent tumors useful or does it confound the patient-matched analysis?

**Response: **Thank you for this insightful question. While we have been exploring both approaches to analyze the data, we also realized that while the bulk comparison provides an initial perspective on metabolic changes throughout glioblastoma progression, it may potentially confound patient-matched analyses. Moving forward, we will focus on patient-matched analyses to pinpoint precise metabolic alterations associated with recurrence within individuals. This approach aims to uncover unique metabolic profiles that play a role in tumor progression and resistance to treatment. We listed this in the limitation section.

1. Interpretation of the 1 plasma sample that showed similar metabolic profiles is premature. The authors’ conclusion that this sample may be evidence of an aggressive signature seems unwarranted as it would suggest that no metabolic alterations at the plasma level leads to early recurrence and patient death. Alternatively, the data may point to a temporal effect of plasma analysis, where in early recurrence, only a short time has elapsed and thus plasma levels are more similar. If this were true, additional analysis would be needed to detail the duration of time between measurements and may suggest strong variance in plasma level analysis.

**Response: **We agree that the interpretation based on n=1 was premature. We have revised the interpretation in the discussion section according to the reviewers' suggestions.

 MINOR COMMENTS:

1. There are several reports within recent years that have investigated the metabolome in GBM. A more exhaustive survey of the literature is needed.

**Response: **We have conducted additional literature reviews and incorporated new references into both the introduction and discussion sections.

1. Figure 1D lists N-alpha-methylhistamine twice

**Response: **We have removed the repeated graph.
